# Correlation of *Streptococcus agalactiae* concentration on immune system and effective dose of inactivated vaccine for Chitralada 3 strain Nile tilapia (*Oreochromis niloticus*) in Thailand

**DOI:** 10.1186/s12917-023-03835-6

**Published:** 2023-12-11

**Authors:** Thanakorn Khunrang, Chettupon Pooljun, Suwit Wuthisuthimethavee

**Affiliations:** 1 School of Agricultural Technology and Food Industry, Walailak University, Thasala District, Nakhon Si Thammarat, 80161 Thailand; 2 Akkhraratchakumari Veterinary College, Walailak University, Thasala District, Nakhon Si Thammarat, 80161 Thailand; 3 Center of Excellence for Aquaculture Technology and Innovation, Walailak University, Thasala District, Nakhon Si Thammarat, 80161 Thailand; 4 Research Center on One Health, Walailak University, Thasala District, Nakhon Si Thammarat, 80161 Thailand

**Keywords:** Immunoglobulin gene expression, *Streptococcus agalactiae* concentration, Tilapia vaccine

## Abstract

The main pathogen in the Nile tilapia (*Oreochromis niloticus*) culture, *Streptococcus agalactiae*, causes economic harm. Infected fish’s immune systems worked to eliminate of the infection. This study demonstrated the effect of different bacterial concentrations on tilapia immunity and optimal vaccine concentration to induce immunity in Nile tilapia. The experiment was performed at 10^2^, 10^4^, 10^6^, 10^8^, and 10^10^ CFU/fish of *S. agalactiae* compared with the control (PBS) through intraperitoneal injection for 72 h. Fish that survived employed to gather blood, and immune responses were assessed through measures of the survival rate include blood smears, antibody titers, and immunoglobulin gene expression. The vaccine experiment investigated formalin-inactivated *S. agalactiae* vaccination and administered *S. agalactiae* injections for 14 days. The statistic revealed a significant difference (*p* < 0.05) in the 10^8^ and 10^10^ CFU/fish injections with high survival rates (62.22% and 53.33%, respectively). Immunoglobulin gene expression was highly represented in the 10^10^ CFU/fish injection; antibody titers were significantly improved from the control group, and antibody levels were high in the 10^10^ CFU/fish injection. The analysis of blood cell types using the blood smear method revealed a progressive increase in leucocytes, particularly lymphocytes, neutrophils, and monocytes, in the treatment group compared to the control group. Moreover, the erythrocyte/leucocyte ratio decreased significantly in response to the high bacterial injection, indicating an increase in leucocytes. Conversely, the erythrocyte level stayed ed within at the 7.03–9.70 × 10^2^ cell/ml and shown no significant difference (*p* > 0.05). The lymphocytes were almost two-fold in 10^10^ CFU/fish compared to 10^8^ CFU/fish. As depicted in the lowest concentration of 10^6^ CFU/fish, the vaccine performance had a high relative percent survival (RPS) at 86.67%. This research suggested that the tilapia infected with high *S. agalactiae* concentrations did not affect the mortality of the tilapia, and vaccine concentration was effective in 10^6^ CFU/fish.

## Introduction

In Thailand, Chitralada 3 tilapia is a strain of tilapia developed from GIFT (Genetic Improvement of Farmed Tilapia), a Philippine ICLARM unit’s fifth generation (tilapia GIFT has traditional Thai Chitralada species mixed with it). Since breeding via the group selection approach was said to have been successful most recently in 2007, “Chitralada 3” has been consistently bred. Noteworthy for its impressive yield, excellent survival rate, and robust growth, “Chitralada 3” is characterized by its small, thick, dense, and highly meaty heads [1].

Streptococcosis, induced by a group B *Streptococcus agalactiae*, stands out as one of the most detrimental bacterial infections affecting Chitralada 3 strain Nile tilapia. This infection has the potential to result in a mortality rate as high as 90% [2, 3]. The bacteria generate the streptolysin S and O for blood hydrolysis and adhesion of cell surfaces, protect lysozyme, and replicate in serum or blood into target organs [4–6]. An infected fish exhibits external signs through abnormal behavior (swirling behavior, lethargy, bent bodies, and disorientation) and eye lesions (endophthalmia or exophthalmia), abscesses, skin hemorrhages around the mouth or at the base of the fin, and ascites [7]. The clinical signs and lesions effects of *S. agalactiae* infection include septicemia, hemorrhages, and inflammation in the liver, spleen, kidney, heart, brain, eye, intestinal tract, and peritoneum. Adhesions to the peritoneal cavity occur in severe infections, resulting in high mortality and severe economic damage. The epidemiology of this disease is caused by stress conditions (high water temperature, suboptimal oxygen, and overcrowding), horizontal transmission from feces, bacteria via lesions, and weakfish [8]. Accordingly, several antibiotic products and probiotics have been used to control this disease, risking beneficial bacteria equivalence often administered through food and environmental conditions. More environmentally friendly methods must be developed to relieve the problem, and the *S. agalactiae* vaccine is an exciting option for amendments to this prevalent pandemic [9].

While *S. agalactiae* attacks the tilapia by leading to immune response as a pathogen exposure response, two different parts exist in the immune system: innate and acquired immunity with cell-mediated and humoral responses. The innate immune system functions as a physical barrier, encompassing elements such as integumentary system, skin, scales, slime and chemicals defenses. Subsequently, it initiates a generalized response characterized by inflammation, opsonization, and phagocytosis. Macrophages and nonspecific cytotoxic cells serve as effectors within this system. In contrast, acquired immunity (AC) involves specific responses to antigen molecules, with B-cell, a subtype of lymphocytes, generating immunoglobulins or antibodies. T-cells and regulator cells acts as effectors in the context of acquired immunity [10].

Immunoglobulin M (IgM), a classical antibody isotype found in most vertebrates, plays a critical role in the host immune response, performing a variety of functions such as neutralizing specific antigens and activating the complement system [11], agglutination, binding of mannose binding lectin [12], and mediating cellular cytotoxicity [13]. IgM serves as the primary antibody produced in response to antigens and represents the initial antibody isotype to emerge during ontogeny.

IgM is classified as the primordial immunoglobulin of the adaptive immune response and is found in monomeric and tetrameric forms in circulating blood [14]. IgM can exist in 2 forms, sIgM and membrane-bound (mIgM), which are generated via alternative RNA splicing of the primary transcript of the μ gene [15]. sIgM consists of the variable region and 4 constant domains in the heavy chain, whereas mIgM contains variable region, 3 constant domains and 2 additional transmembrane domains (TM1 and TM2) and acts as a B cell receptor for initial antigen binding [16]. Together with innate immunity factors, it offers the initial line of defense against microbial infection. Until date, the immunoglobulin heavy (IGH) chain gene complex has encoded three primary types of immunoglobulins (Igs) in teleost: IgM [17], IgD [18], and IgT/IgZ. The considerable increase in IgM expression in Nile tilapia following bacterial challenge [13].

Despite the reality that tilapia culture frequently uses S. agalactiae vaccinations. These vaccinations come in several forms, such as DNA, recombinant, live attenuated, and whole-cell inactivated vaccines; each has a unique preparation and bacterial specificity [19, 20]. However, formalin-killed cell vaccine (FKCV) is a whole-cell inactivated vaccine successfully developed for tilapia and comprises the whole cell and a subunit of dead bacterial cells [21]. The intraperitoneal administration of the formalin-killed vaccine in tilapia has been extensively shown to produce highly effective outcomes, as evidenced by the relative percent survival (RPS). The FKC vaccine involves adaptive immunity to release immunoglobulin M (IgM) for primary defense with immunological memory. Therefore, the *S. agalactiae* vaccination is an extensively recognized process to prevent streptococcosis outbreaks and reduce fish mortality in the tilapia industry [22–24].

This research aimed to compare the immunoglobulin gene expression immune responses of tilapia infected with different *S. agalactiae* (serotype Ia) concentrations and effective dose of FKC vaccine as an alternative prevention approach in tilapia against *S. agalactiae*.

## Materials and methods

### ***Streptococcus agalactiae*** isotype Ia

*S. agalactiae* (serotype Ia) obtained from Kidchakan Supamattaya Aquatic Animal Health Research Center, Department of Aquatic Science, Faculty of Natural Resources, Prince of Songkhla University, Thailand. Multiplex PCR molecular serotyping was used to identify and validate the *S. agalactiae* serotype [25]. Prior to the experimental challenge, the bacterial isolation was passage through the fish with 0.1 ml of 108 CFU/ml by intraperitoneal (IP) injection twice to enhance their virulence post storage (-80^o^C). The bacteria were recovered from the blood of freshly dead fish and were cultured on Trypticase soy agar (TSA) media with 5% sheep blood at 37^o^C for 18 h. The activated bacteria were identified using specific primers (Forward: GAGTTTGATCATGGCTCAG and Reverse: ACCAACATGTGTTAATTACTC) for *S. agalactiae* [26]. Briefly, the amplification condition included a holding step at 95 ^o^C for 15 min, 35 cycles of denaturation at 95 ^o^C for 30 s, annealing at 55 ^o^C for 30 s, extension at 72 ^o^C for 1 min and finally extension at 72 ^o^C for 5 min. The PCR product was investigated by using 1% agarose gel electrophoresis using 1× TBE buffer and 100 bp DNA ladder ready to load (Solis biodyne, Tartu, Estonia). Following bacterial confirmation, the bacteria were diluted in phosphate-buffered saline, PBS (Calbiochem, USA). The optical density (OD) at 600 nm, set at 1.00 (equivalent to 2.5 × 10^12^ CFU), was utilized to formulate the treatment [27].

### Experimental tilapia

Healthy Nile tilapia of chitralada 3’s strain (approximately 20 g weight) was obtained from a private fish farm in Nakhon Si Thammarat province, Thailand. They were acclimated for ten days in the experimental zone in 500-liter tanks with a density of 100 fish per tank with aeration. Tanks were supplied with flow-through dechlorinated tap water and air stones to maintain desired water temperature, dissolved oxygen (DO) and pH levels. Water quality (temperature, DO, and pH) was measured daily and maintained at 28–30 °C for water temperature, > 4 ppm for DO, and pH 7–8. During the acclimation period, the fish were fed with a commercial diet (THAILUXE® comprised 40.17% protein, 4.32% lipid, 18.74% ash, and 15.84% moisture) twice daily at 9.00 am and 4.00 pm at 5% of body weight and maintained and handled according to Institutional Animal Care and Use Committee–approved guidelines. Five tilapias were randomly tested for *S. agalactiae* infection using PCR to ensure they were free of infection before the experiments.

Prior to investigation of severity, blood puncture, vaccination performance, the fish were anesthetized with buffered TMS (MS-222, Sigma, Cat: A5040). The experiment was conducted in strict accordance with the guidelines of the Animal Care and Use Committee of Walailak University and the regulations governing the use of animals in experimentation, and the protocol was approved by the Animal Ethics Committee, Walailak University (Protocol No. WU-AICUC-63-042) and all of the experiments were conducted in accordance with the guidelines and regulations of the Management and Use of Laboratory Animals of the Animal Ethics Committee, Walailak University. All efforts were made to minimize suffering and stress. Any fish that showed signs of disease or abnormal behavior (lethargy, bloating, disoriented swimming) or moribund was euthanized by using the hypothermal shock method in accordance with the guidelines outlined in the American Veterinary Medical Association (AVMA) Guidelines for the Euthanasia of Animals: 2020 Edition. In brief, the fish were gently captured and subsequently immersed in a container filled with cold water (2–4 °C) for 5–10 min, inducing loss of consciousness and eventual euthanasia. Following the prescribed time interval, the cessation of opercular movement was monitored to confirm euthanasia. Dead fish were then securely placed in plastic bags and frozen for further study. At all sampling times, fish were almost completely exsanguinated during blood collection and also euthanized with the hypothermal shock method.

### Investigation of severity (pathogenicity)

The acclimated Nile tilapia fish was used to determine mortality after *S. agalactiae* infection at different bacterial concentrations. The fish were randomly divided into six groups with four replicates per group and 15 fish for each replicate, and they were held in an 80-liter plastic tank with aeration. One group was designated as the control (PBS injection), and the others were inoculated with five different concentrations (10^2^, 10^4^, 10^6^, 10^8^, or 10^10^ CFU/fish) of the *S. agalactiae* stock. The fish in the challenge groups were intraperitoneally injected with 0.2 ml of *S. agalactiae* dilutions which appropriate volume for 20–30 g Nile tilapia. In contrast, the fish in the control group were injected with 0.2 ml of PBS. During the experimental period, the fish were fed twice daily, and the water was changed every three days. Cumulative mortality, clinical signs were observed and recorded twice daily after challenge for 14 days. To verify the presence of *S. agalactiae* infection in the dead or moribund and the surviving fish, PCR tests using F1 (GAGTTTGATCATGGCTCAG) and IMOD (ACCAACATG TGTTAATTACTC) were conducted [26]. Water quality was measured daily and maintained at 28–30 °C for water temperature > 4 ppm for DO and pH 7–8. During the acclimation period, the fish were fed with a commercial diet twice daily at 9.00 am and 4.00 pm at 5% of body weight.

In this challenge test, mortality data following bacterial infection were derived from three replicates within each group. The remaining replicate in each group was utilized to investigate the immune response through fish replication.

### Blood puncture and smear

After 72 h post-challenge, 500 to 1,000 μl of blood was collected from the individual caudal vein of ten infected fish from each group to measure antibody titers, gene expression, and blood smears. One drop of blood was spotted on a cleaned slide, and a glass cover equalized it. The blood slide was fixed with methanol by dipping 3–4 times and stained with commercial Dip Quick Stain (M&P IMPEX, Thailand). The fixed slide was dipped in eosin solution for ten seconds and cleaned with distilled water. Afterward, it was dropped in methylene blue for ten seconds and rewashed with water. The stained slide was air-dried for 400x microscopic observation. The erythrocyte, leucocyte, and thrombocyte counts were determined from the blood.

### Agglutination techniques

For bacterial agglutination, 200 μl of blood was used following modified agglutination methods [27]. Blood was collected in 200 μl samples for bacterial agglutination. The blood was centrifuged at 6,000 xg^− 1^ for 5 min to separate the serum, and serial dilution of the serum was started at 1:10 (10 μl serum and 90 μl 1x PBS). Two-fold serial serum dilution was prepared in 96 well-round bottom microtiter plates (Nunc™ 96-Well Polystyrene Round Bottom Microwell Plates Thermo Scientific™). The remaining well plates were dropped in 50 μl of PBS. The serum was diluted until 1:320. The 50 μl of *S. agalactiae* at OD 1.0 was added to the well. In the experiment, the serum mixed with PBS and bacteria combined with PBS were used as controls. The plates were coned with a plastic cover and incubated at room temperature for 18 h. The endpoint of cell agglutination was observed as the last serum dilution compared to the positive control. The visible agglutination was reported as log 2 of the previous serum.

### Immunoglobulin gene expression analysis

The remaining blood was dropped into a 1,500 μl Eppendorf tube with 3.2% sodium citrate in a 9:1 proportion. The total blood was centrifuged at 6,000 xg^− 1^ for five min for plasma removal and RNA isolation, following the GENEzol™ reagent (Geneaid) procedure. A NanoDrop 2000c spectrophotometer measured RNA quantity and quality (Thermo Fisher Scientific, Wilmington, DE, USA) at 260 and 280 nm wavelengths. The RNA sample was equally diluted to 500 μg/μl and was combined in each treatment to synthesize the cDNA with the iScript™ cDNA Synthesis Kit protocol (BIO-RAD, USA). The cDNA quality was examined with elongation factor 1, housekeeping gene (Forward: GCACGCTCTGCTGGCCTTT, Reverse: GCGCTCAATCTTCCATCCC), and immunoglobulin gene (Forward: GGGAAGATGAGGAAGGAAATGA, Reverse: GTTTTACCCCCCTGGTCCAT), producing PCR product of 250 and 120 bp, respectively [28]. The PCR reaction involved holding at 95^o^C for 15 min, 35 cycles of denaturation at 95^o^C for 30 s, annealing at 60^o^C for 30 s, extension at 72^o^C for 30 s, and a final extension at 72^o^C for five min. The cDNA quality was examined via PCR products using 1% agarose gel electrophoresis. Real-time PCR was employed to examine the expression of the immunoglobulin gene using the ABI 7300 Real-Time PCR System from Applied Biosystems. The amplified gene in 10 μl comprised 1.0 μl of cDNA, 2.0 μl of master mix (5× HOT FIREpol Probe qPCR Mix Plus (ROXX) (Solis Biodyne, Tartu, Estonia)), 0.25 μl of each primer (10 μM), and 6.5 μl of nuclease-free water. The cycling continued for 15 min at 95 °C, followed by 40 cycles at 95 °C for 30 s, 60 °C for 30 s, and 72^o^C for 30 s. The expression level was calculated according to the 2^−ΔΔCT^ method [29].

### Formalin-inactivated ***Streptococcus agalactiae*** vaccine preparation

Trypticase soy broth (TSB) was used to culture active *S. agalactiae* and incubated at 37^o^C in a shaker for 36 h to a final concentration of 1.819 at OD 600 nm. The incubation was conducted at 37^o^C for 48 h and centrifuged to gather the cells before washing with sterile PBS five times. The bacteria were collected by centrifugation at 3,500 ×g for 10 min, and the cell pellets were washed with 1x PBS five times. The PBS was diluted in the bacterial solution until OD 1.000 with a total volume record. The serial dilution technique counted the bacteria cells and combined them again by adding formaldehyde at 0.5% in the solution. The vaccine underwent verification to ensure there was no contamination, utilizing TSA agar and stored at 4^o^C until use.

### Vaccination performance

Tilapias were divided into seven groups for testing with *S. agalactiae* vaccination. In each group, 40 fish were injected with formalin-killed *S. agalactiae* at 10^2^, 10^4^, 10^6^, 10^8^, or 10^10^ CFU/fish concentrations. After 14 days of stimulation, the tilapias were injected with 200 μl of *S. agalactiae* (2.17 × 10^7^ CFU/fish). Conversely, the positive control was injected with active *S. agalactiae* and the negative control with PBS. Cumulative mortality was recorded for the 14-day trial to calculate the relative percent survival as follows: RPS = [1 - (% mortality of vaccinated fish) × (% mortality of control fish)] × 100.

### Statistical analysis

The software SPSS Statistics for Windows (Version 20.0) was utilized to analyze cumulative mortality, survival rate, antibody titers, blood cell and immunoglobulin gene expression data through one-way analysis of variance (ANOVA) and Duncan’s multiple range test. The results were presented in triplicate, depicting mean and standard deviation values. Significant differences among treatments were determined at *p* < 0.05.

## Results

### Challenge test of ***Streptococcus agalactiae*** for tilapia

**Fig. 1:**
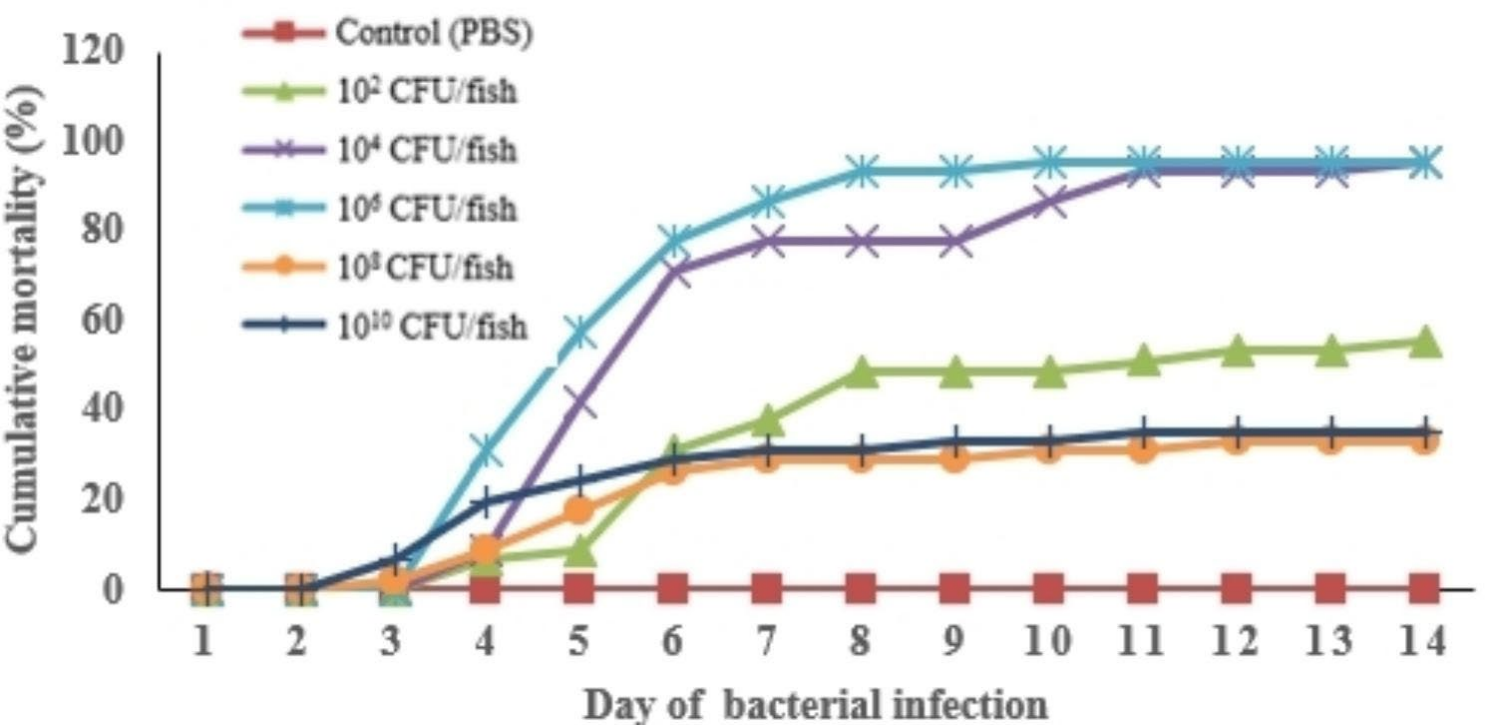
Cumulative mortality of Nile tilapia under different concentrations of *S. agalactiae* injection. Each value is displayed as mean ± SD (n = 3)

The challenge test was estimated for 14 days. After infection with *S. agalactiae*, the death of tilapia occurred only around 3–12 days (Fig. 1). The cumulative mortality of tilapia was 25.08%, 21.75%, 65.56%, 58.41%, and 31.75% within 14 days post-infection with *S. agalactiae*, at doses of 10^10^, 10^8^, 10^6^, 10^4^ and 10^2^ CFU/fish, respectively (Fig. 1). The accumulative mortality shown in Fig. 1 indicates elevated mortality from Days 4 to 7 post-infection, with survival observed on the 6th day following injections of 10^4^ and 10^6^ CFU/fish, as detailed in Table 1.

**Table 1:** The survival rate following injections of varying concentrations of *S. agalactiae* in Nile tilapia

Treatment	Cumulative mortality (%)	The survival rate of different *S. agalactiae* concentrated injections (%)
Control (PBS)	0.00 ± 0.00^c^	100.00 ± 00^a^
10^4^ CFU/fish	58.41 ± 6.29^a^	4.44 ± 3.858^d^
10^6^ CFU/fish	65.56 ± 3.065^a^	6.67 ± 0.00^d^
10^8^ CFU/fish	21.75 ± 4.11^b^	62.22 ± 7.70^ab^
10^10^ CFU/fish	25.08 ± 8.39^b^	53.33 ± 13.33^b^

*The data is presented as mean ± standard deviation (n = 3 fish). Distinct superscript letters within the same row signify statistically significant differences (*p* < 0.05) among the treatments, determined through one-way ANOVA followed by Duncan’s multiple range test

### Serum agglutination titers

**Fig. 2:**
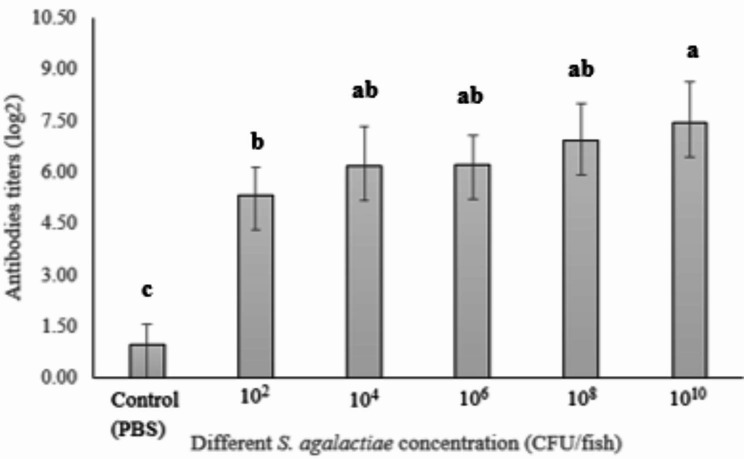
Serum antibody titers in tilapia were evaluated using bacterial agglutination techniques with different concentrations of *S. agalactiae* injection, comparing them to the control (PBS injection). Distinct superscript letters denote statistically significant differences (*p* < 0.05) among the treatments

We observed that the various antibody levels in the tilapia having a significant (*p* < 0.05) difference compared to the control group. Figure 2 presents the antibody titers with the bacterial challenge. The highest titers were shown in 10^10^ CFU/fish of the *S. agalactiae* injection, but no significant (*p* > 0.05) differences were found in 10^4^, 10^6^, and 10^8^ CFU/fish. The 10^2^ CFU/fish injection revealed a higher antibody level than the control group (PBS).

**Fig. 3:**
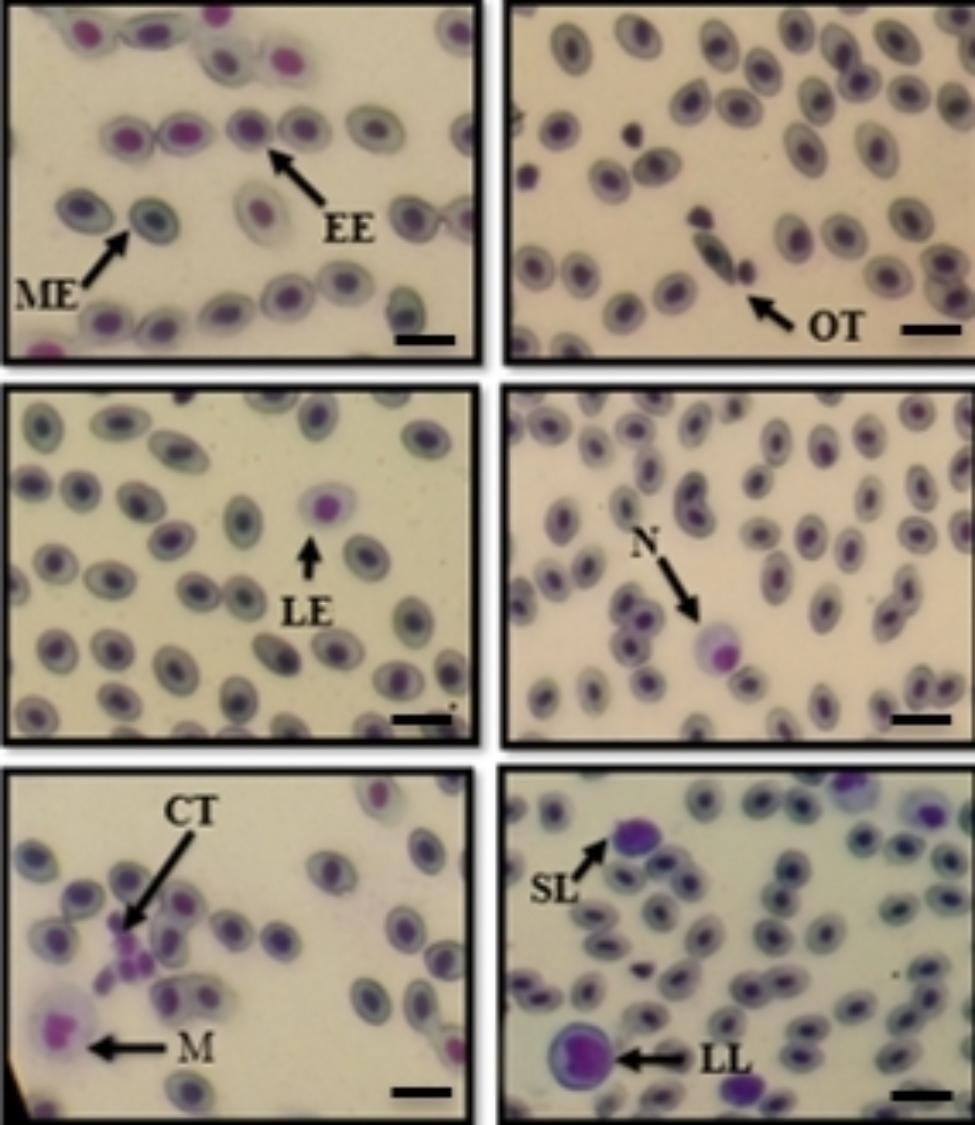
Peripheral blood cell morphology of Nile tilapia on blood smears was performed using light microscopy with 40x. Early immature erythrocytes (EE), Mature erythrocytes (ME), Large immature erythrocytes (LE), Clusters of fragmented thrombocytes (CT), Monocytes (M), Oval thrombocytes (OT), Neutrophils (N), Small lymphocytes (SL), and large lymphocyte (LL). Scale bars = 12.5 μm

**Fig. 4:**
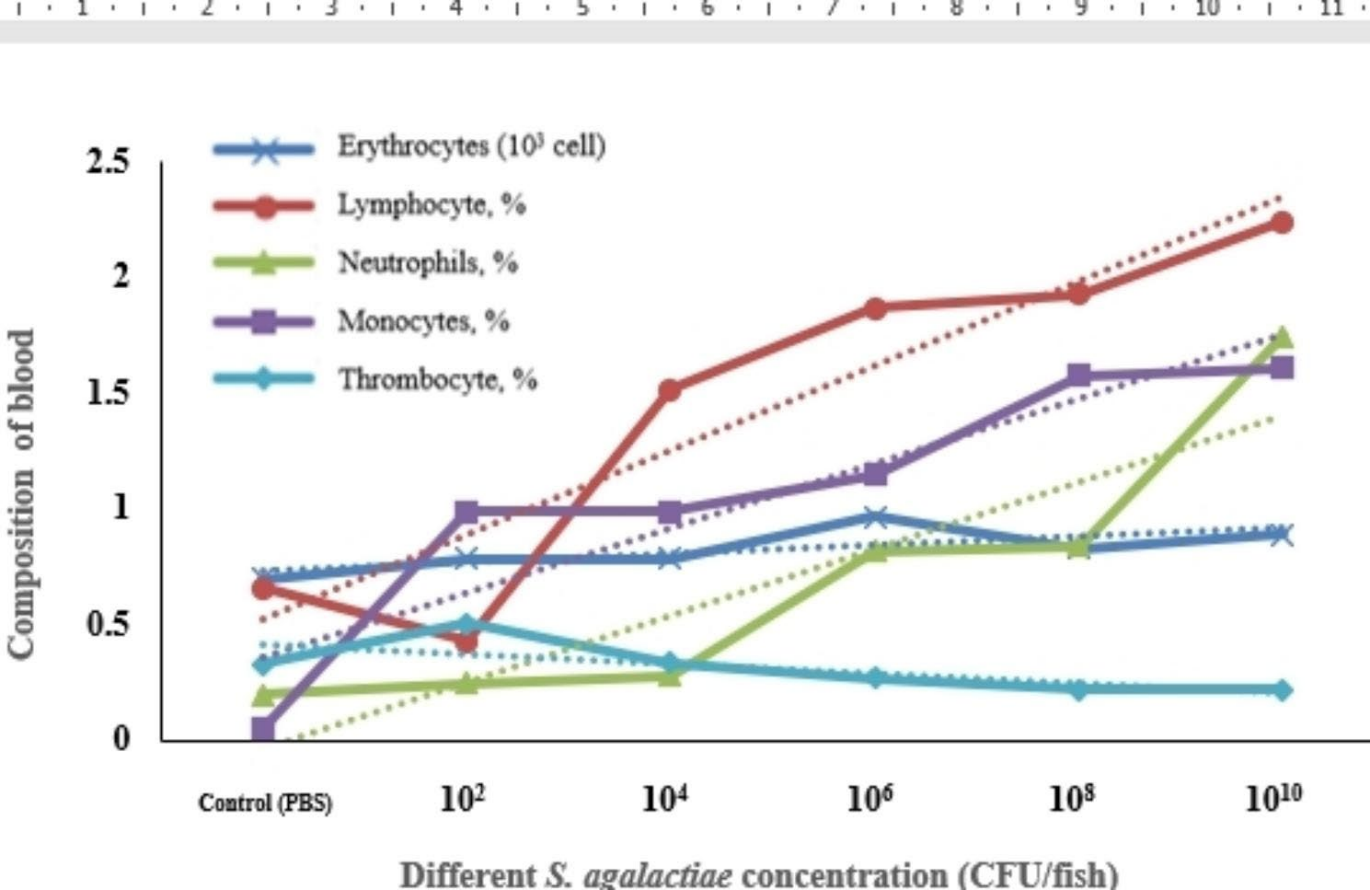
The blood cell composition of Nile tilapia under different concentrations of *S. agalactiae* injection. Each value is presented as mean ± SD (n = 10)

**Fig. 5:**
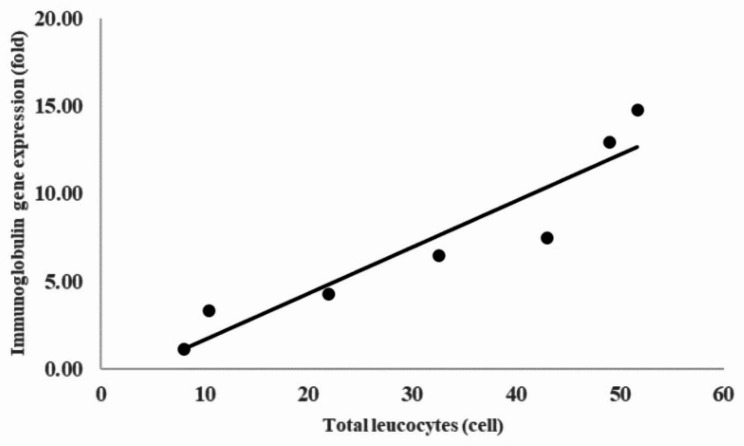
The correlation between immunoglobulin gene expression and total leucocyte cells in Nile tilapia. The data is presented as mean ± SD from triplicate samples

The total blood count results by the blood smear technique variously depicted erythrocytes and white blood cells (WBCs) of each type (Fig. 3). Increased bacterial concentration caused WBCs accumulation. Therefore, statistical analysis demonstrated a significant (*p* < 0.05) difference between the control and injection treatments. The high bacterial inoculum concentration impacted the increase in lymphocyte, neutrophil, and monocyte counts (Fig. 4). The control was revealed to have total lymphocytes at 0.66%, while the 10^2^ CFU/fish injection had total lymphocytes at 2.24%, with significant differences between the two groups (Table 2). Figure 5 depicts a positive correlation between the expression of the immunoglobulin gene and the total leucocyte count.

**Table 2:** Percent of erythrocytes/leucocytes of different concentrations of the *S. agalactiae* injection compared with the control (PBS) under the blood smear technique at 40x microscopic performance for Nile tilapia

Experimental variants	Treatments					
	Control (PBS)	10^2^	10^4^	10^6^	10^8^	10^10^
Erythrocytes (x10^3^ cell)	0.70 ± 0.27	0.79 ± 0.11	0.79 ± 0.29	0.97 ± 0.43	0.83 ± 0.47	0.89 ± 0.24
Lymphocytes, %	0.66 ± 0.56^b^	0.43 ± 0.34^b^	1.52 ± 1.01^a^	1.87 ± 0.83 ^a^	1.93 ± 0.73 ^a^	2.24 ± 1.61 ^a^
Neutrophils, %	0.20 ± 0.18^c^	0.25 ± 0.15^c^	0.28 ± 0.17^c^	0.82 ± 0.81^b^	0.84 ± 0.56^b^	1.75 ± 0.50^a^
Monocytes, %	0.15 ± 0.19^b^	0.99 ± 0.47^a^	0.99 ± 0.73^a^	1.15 ± 0.94^a^	1.58 ± 0.83^a^	1.61 ± 2.17^a^
Thrombocytes, %	0.33 ± 0.19^ab^	0.51 ± 0.39^b^	0.34 ± 0.16^ab^	0.27 ± 0.17^a^	0.22 ± 0.14^a^	0.22 ± 0.15^a^

*The data is presented as mean ± standard deviation (n = 10 fish). Distinct superscript letters in the same row signify statistically significant difference (*p* < 0.05) among the treatments, determined through one-way ANOVA, followed by Duncan’s multiple range test

### Immunoglobulin gene expression levels

**Fig. 6:**
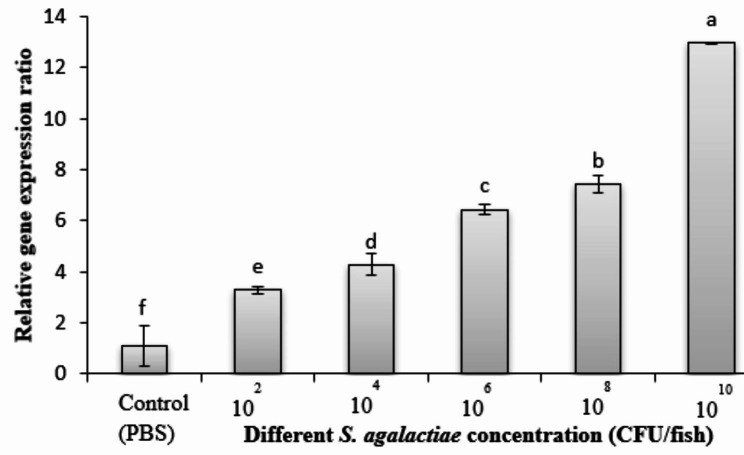
The relative expression of the immunoglobulin gene in tilapia blood under different concentrations of the *S. agalactiae* injection compared with the control (PBS injection). Different superscript letters indicate statistically significant (*p* < 0.05) differences among the treatments

Figure 6 depicted immunoglobulin gene expression of the tilapia blood after injection with different concentrations of bacteria. The expression gradually increased in the bacteria injection groups more than in the control group injected with PBS. However, the expression levels of the immunoglobulin gene in fish groups injected with 10^4^-10^8^ CFU/fish did not reveal a significant difference (*p* > 0.05) among the groups. The higher significance levels (*p* < 0.05) were distinguished in the 10^10^ CFU/fish injection. The expression of 10^2^ CFU/fish also did not significantly (*p* > 0.05) differ from the control groups.

### Vaccine efficacy

As depicted in Table 3, tilapia immunized with the *S. agalactiae* vaccine was demonstrated in 10^6^ CFU/fish; the survival rate was 86.67%. The vaccination with the 10^2^ and 10^4^ CFU/fish illustrated that no fish survived after the *S. agalactiae* injection. The RPSs were highly demonstrated in 10^6^ CFU/fish vaccination, but statistically significant (*p* > 0.05) differences did not occur with 10^8^ and 10^10^ CFU/fish. The corresponding RPSs were 86.67, 83.33, and 76.67%, respectively.

**Table 3:** Survival rate and relative percent survival of vaccinated performance of Nile tilapia

Treatment	Survival rate of vaccine experiment (%)	% RPS of vaccine test
Control (PBS)	100.00 ± 0.00^a^	-
Control (S. agalactiae)	0.00 ± 0.00^c^	-
10^2^ CFU/fish	0.00 ± 0.00^c^	0.00 ± 0.00c
10^4^ CFU/fish	0.00 ± 0.00^c^	0.00 ± 0.00c
10^6^ CFU/fish	86.67 ± 15.26^a^	86.67 ± 15.28^a^
10^8^ CFU/fish	83.33 ± 5.77^a^	83.33 ± 5.77^a^
10^10^ CFU/fish	86.67 ± 5.77^a^	76.67 ± 5.77^a^

*The data is presented as mean ± standard deviation (n = 3 fish). Distinct superscript letters in the same row signify statistically significant difference (*p* < 0.05) among the treatments, determined through one-way ANOVA, followed by Duncan’s multiple range test.

## Discussion

*S. agalactiae* is one of the most important pathogens and causes high mortality in Nile tilapia. The immune responses of Nile tilapia infected *S. agalactiae* in this study has led to the understanding of a specific immune responses including antibody levels, hematological parameters, and immunoglobulin gene expression of Nile tilapia when infected with *S. agalactiae*. After challenge, the results indicate that the challenge with *S. agalactiae* can cause clinical signs and lesions in Nile tilapia, when the bacteria were delivered intramuscularly inoculation, mortality was initially observed within 72 h. A linear dose response was not seen. This result suggests that the elevated bacterial concentration did not lead to disease, as evidenced by higher tilapia survival rates at 10^8^ and 10^10^ CFU/fish compared to 10^2^, 10^4^, and 10^6^ CFU/fish. The results also showed that a high concentration of bacteria could activate the adaptive immune system concerning the expression of the IgM gene. The number of lymphocytes increased in the high bacterial concentration treatments. The decrease of mortality at higher dilution of *S. agalactiae* was possibly due to factors effecting immunological tolerance, especially the increasing of IgM level which the expression of the IgM gene in the blood demonstrated that the high bacteria injection resulted in extremely high levels of expression. The IgM gene relates to acquired immunological response antibodies with a role in bacterial defense [13, 30]. Additionally, the IgM gene is expressed in various tissues, including the head kidney, spleen, intestine, skin, and gill. Vital tissues for producing antibodies for B and T cells in bony fish, the head kidney, and spleen had the highest IgM expression. Moreover, the IgM gene was detected in the skin and intestine’s mucosal immune system [31]. The mucosal immune system is the first line of defense against pathogen invasion and is susceptible to bacterial stimulation of phagocytosis via the mucosal surface [13]. The experiment detected bacteria from the blood throughout the target organs (brain, liver, spleen, and kidney). The IgM heavy chain gene was thoroughly expressed in all tested tissues, but higher levels were expressed in the peripheral blood leukocyte. Thus, the blood sample was the appropriate example of IgM gene expression to reduce the experimental fish mortality from tissue collection [32].

Antibody titers demonstrated that varying bacterial concentrations increased the augmentation of antibody levels; however, no statistically significant differences (*p* > 0.05) existed among 10^4^–10^10^ CFU/fish. The lymphocyte and immunoglobulin produced antibodies with diverse functions in serum and mucus, and they participated in the B cell surface complex and antigen signaling function [33, 34]. The IgM titer should increase bacterial activation based on the quantities of bacteria found in the experiment. The dosages of bacteria also affected the immune protection response [35] The results exhibited a strong relationship between bacterial concentrations and antibody levels.

The number of leucocytes, observed by the blood smear technique, increased directly with the quantities of bacteria, according to the study. We determined the increments of lymphocytes, neutrophils, and monocyte counts. Lymphocytes, thrombocytes, monocytes, granulocytes, and nonspecific cytotoxic cells existed among the white blood cells. Leucocytes contributed to functional retardation and pathogen elimination via the immune system during pathogen assessment, commonly suggesting a contrary relationship with the fish’s condition or health [36]. Individually infected fish produced many leucocytes for antibody generation and phagocytosis. Lymphocytes appear to be made by the thymus, spleen, and kidney. The primary functions of antibody production and phagocytic activity induce the macrophage with this efficacy, where the antibodies were proportional to the number of lymphocytes [37, 38]. Therefore, excessive lymphocyte increase affects the survival of tilapia [36].

Furthermore, fish thrombocytes were accountable for the precursors of blood clotting in circulating fluids. Fish thrombocytes come in various shapes (oval, spindle, spiked, and cluster of fragmented thrombocytes), and every thrombocyte was sharp on blood smear slides [37]. A small proportion of the white blood cell population comprises monocytes or macrophages. Thought to originate in the kidney, they are competent in killing a category of pathogens and bacteria. In addition, neutrophils are one type of granulocyte elucidating up to 25% of the overall leukocyte population. Teleost produces granulocytes in the kidney and spleen [39]. Neutrophils migrate to the site of bacterial infection, where they may be phagocytic or bactericidal and frequently correlate with stress [40].

Moreover, vaccination demonstrated the effectiveness of *S. agalactiae* resistance 14 days following the induction of immunity. The intraperitoneally vaccinated injection with the whole-cell inactivated *S. agalactiae* vaccine resulted in high survival at 10^6^ CFU/fish concentration and no significant difference (*p* > 0.05) at 10^8^ and 10^10^ CFU/fish. In contrast, no fish survived in 10^2^ and 10^4^ CFU/fish after the *S. agalactiae* injection for 14 days of observation. Through intraperitoneal injection, formalin-killed vaccines exhibited excellent protection and a high relative percent survival (75–100%). Although no finding regarding the immune system existed, the fish experiment can be seen as a sign of success [41].

## Conclusion

Our experiment has demonstrated the effect of highly concentrated *S. agalactiae* injections on immunological induction and survival, including an increase in the immunoglobulin gene expression and antibodies and an increase in leucocytes. The research indicated that injections of highly concentrated *S. agalactiae* reduce virulence and reverse immunological activation. Therefore, different vaccination concentrations had varying effects. We advocate vaccinating juvenile tilapia with 10^6^ CFU/fish of *S. agalactiae* vaccination to reduce *S. agalactiae* outbreaks in the tilapia farm industry and for convenience. Furthermore, we propose vaccination as an alternative method to safeguard tilapia population against *S. agalactiae* infection.

## Data Availability

The authors declare that they do not have any shared data available.
